# Depression and 24 gastrointestinal diseases: a Mendelian randomization study

**DOI:** 10.1038/s41398-023-02459-6

**Published:** 2023-05-04

**Authors:** Xixian Ruan, Jie Chen, Yuhao Sun, Yao Zhang, Jianhui Zhao, Xiaoyan Wang, Xue Li, Shuai Yuan, Susanna C. Larsson

**Affiliations:** 1 Department of Gastroenterology, The Third Xiangya Hospital, Central South University, Changsha, China; 2 Department of Big Data in Health Science, School of Public Health and The Second Affiliated Hospital, Zhejiang University School of Medicine, Hangzhou, Zhejiang China; 3 Department of Gastroenterology, Ruijin Hospital, Shanghai Jiao Tong University School of Medicine, Shanghai, China; 4 Unit of Cardiovascular and Nutritional Epidemiology, Institute of Environmental Medicine, Karolinska Institutet, Stockholm, Sweden; 5 Unit of Medical Epidemiology, Department of Surgical Sciences, Uppsala University, Uppsala, Sweden

**Keywords:** Depression, Clinical genetics

## Abstract

The causality of the association between depression and gastrointestinal diseases is undetermined. We conducted Mendelian randomization (MR) analyses to systematically explore the associations of depression with 24 gastrointestinal diseases. Independent genetic variants associated with depression at the genome-wide significance level were selected as instrumental variables. Genetic associations with 24 gastrointestinal diseases were obtained from the UK Biobank study, the FinnGen study, and large consortia. Multivariable MR analysis was conducted to explore the mediation effects of body mass index, cigarette smoking, and type 2 diabetes. After multiple-testing corrections, genetic liability to depression was associated with an increased risk of irritable bowel syndrome, non-alcohol fatty liver disease, alcoholic liver disease, gastroesophageal reflux, chronic pancreatitis, duodenal ulcer, chronic gastritis, gastric ulcer, diverticular disease, cholelithiasis, acute pancreatitis, and ulcerative colitis. For the causal effect of genetic liability to depression on non-alcoholic fatty liver disease, a substantial proportion was mediated by body mass index. Genetic predisposition to smoking initiation mediated half of effect of depression on acute pancreatitis. This MR study suggests that depression may play a causal role in many gastrointestinal diseases.

## Introduction

Depression is a common and serious mental illness that limits psychosocial functioning and compromises life quality [1]. The prevalence of digestive system disease has been found to be higher in depressive patients compared to the general population [2, 3]. Most observational studies have investigated the role of gastrointestinal disorder in development of depression [4, 5], but limited on the reverse impact. Previous cohort studies found that depression was associated with an increased risk of irritable bowel syndrome [6], gastroesophageal reflux [7], and peptic ulcer [8]. Evidence from the Nurses’ Health Studies also found that self-reported depressive symptoms were associated with an increased risk of Crohn’s disease but not ulcerative colitis [9]; however, an association between new-onset depression and ulcerative colitis was revealed in another study [10]. The inconsistent findings as well as the limitations of observational studies, like residual confounding and reverse causation, hinder the causal assessment of the associations between depression and gastrointestinal diseases.

Mendelian randomization (MR) is a method that employs genetic variants as instrumental variables for the exposure to infer the causality of an exposure-outcome association [11]. Compared to conventional observational studies, MR is by nature less prone to confounding since genetic variants are randomly assorted at conception and, therefore, unrelated to environmental factors. In addition, this method can minimize reverse causation as germline phenotypes cannot be modified by disease status. Although a phenome-wide MR study found some associations of depression with inflammatory and hemorrhagic gastrointestinal diseases [12], the effects of depression on a broad range of gastrointestinal outcomes have not been investigated. Here, we performed an MR study to examine the associations of genetic liability to major depressive disorder with 24 gastrointestinal diseases. To reveal possible mechanistic pathway, we further conducted multivariable MR analysis to examine the mediations of body mass index, tobacco smoking, and type 2 diabetes mellitus.

## Method

**Fig. 1:**
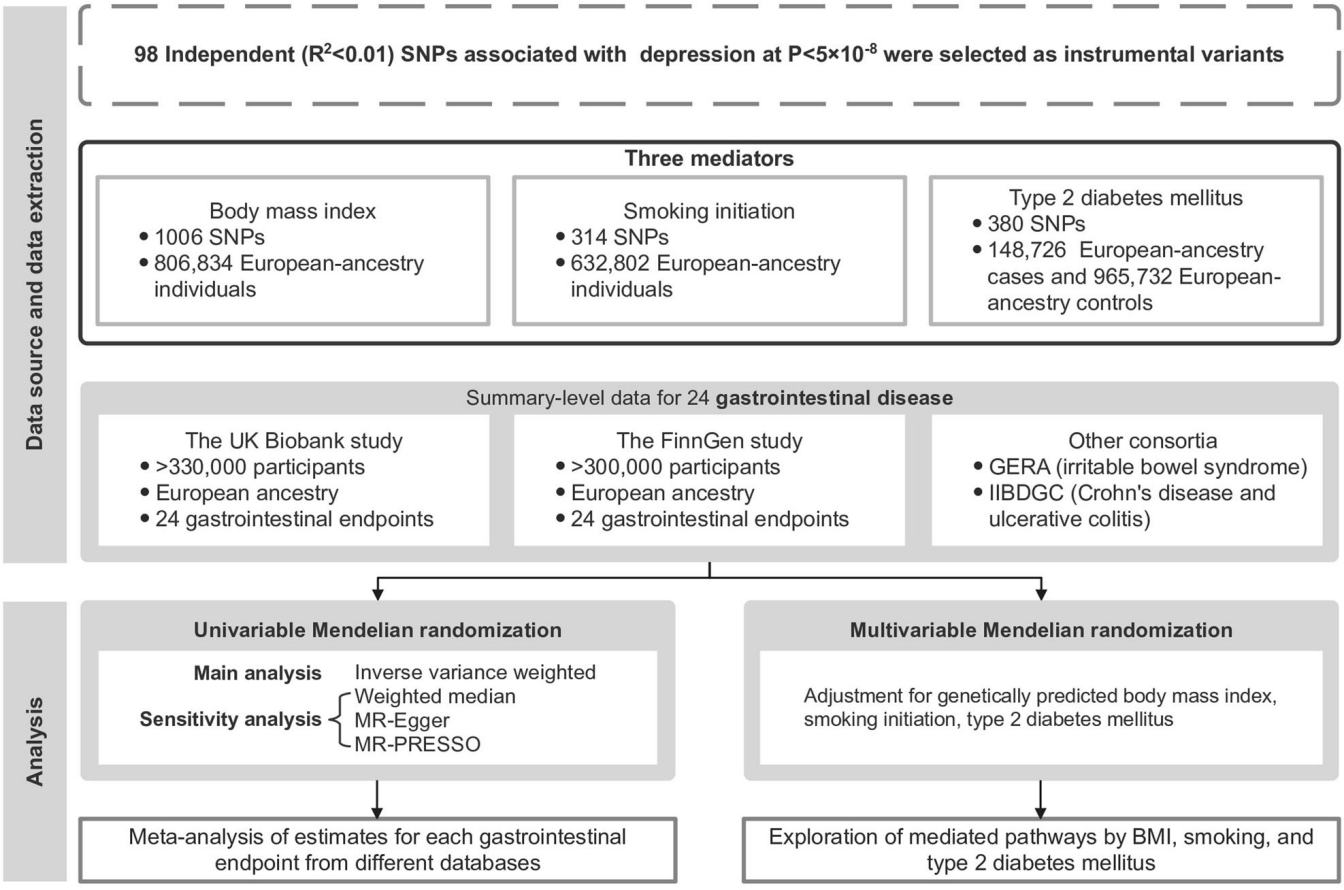
Study design. BMI body mass index, GERA Genetic Epidemiology Research on Aging, IIBDGC International Inflammatory Bowel Disease Genetics Consortium, MR Mendelian randomization, MR-PRESSO Mendelian randomization pleiotropy residual sum and outlier, SNP single nucleotide polymorphisms.

Figure 1 shows the overview design of the study. This MR investigation was based on publicly available genome-wide association study (GWAS) consortia (Table S1). All MR analyses were performed separately in each dataset, including the UK Biobank study [13], the FinnGen study [14], and other large consortia if available. Individual MR estimates for each gastrointestinal endpoint were pooled. Included studies had been approved by corresponding institutional review boards and ethical committees.

### Instrumental variable selection

Genetic instrumental variables for major depressive disorder were extracted from the latest GWAS, which meta-analyzed data in 807,553 individuals (246,363 depressive cases and 561,190 controls predominantly of European ancestry) from the UK Biobank study, 23andMe, and Major Depressive Disorder Working Group of the Psychiatric Genomics Consortium (PGC) [15]. In total, 98 single nucleotide polymorphisms (SNPs) associated with depression at the genome-wide significance threshold (*P* < 5 × 10^−8^) and without linkage disequilibrium (defined as *r*^*2*^ > 0.01) were identified. Detailed information on used SNPs is presented in Table S2. Odds ratio (ORs) and CIs of outcome were scale to per one-unit increase in log odds of liability to depression.

### Gastrointestinal disease data sources

Genetic associations with 24 gastrointestinal diseases were obtained from the UK Biobank study [13], the FinnGen study [14], and two large consortia, including the International Inflammatory Bowel Disease Genetics Consortium (IIBDGC) [16] and Genetic Epidemiology Research on Aging (GERA) [17]. The UK Biobank is a large multicenter cohort study comprising half a million individuals recruited between 2006 and 2010 in the United Kingdom [13]. The database collects information on a wide range of health-related variables, including self-reported information, clinically validated data, and register-based data. Summary statistics of European ancestry in UK Biobank were obtained from GWAS conducted by the Lee lab where the gastrointestinal outcomes were defined by codes of the International Classification of Diseases 9th Revision (ICD-9) and ICD-10. The genetic associations were adjusted for sex, birth year, and the first four genetic principal components. As for the FinnGen study, the latest summary-level genetic data (R7 release) on gastrointestinal diseases were obtained [14]. The FinnGen study involves the collection and analysis of genetic data from over 500,000 participants from the Finnish biobanks, along with their digital health record data from the Care Register for Health Care, and information from the cancer, cause of death, and medication reimbursement registries. The gastrointestinal endpoints were defined by ICD-8, ICD-9, and ICD-10 codes. Genome-wide association analyses were adjusted for sex, age, genetic components, and genotyping batch in FinnGen. Detailed diagnostic codes in UK Biobank and FinnGen are listed in Table S3. We also obtained summary-level data from the IIBDGC [16] for Crohn’s disease (5956 cases and 14,927 controls) and ulcerative colitis (6968 cases and 20,464 controls) and from the GERA for irritable bowel syndrome (3117 cases and 53,520 controls) [17]. Diagnosis of IBD in IIBDGC was based on accepted radiologic, endoscopic, and histopathologic evaluations. GERA used longitudinal electronic health records to obtain clinical information of individuals.

### Data sources for possible mediators

Depression has been associated with body mass index (BMI) [18], cigarette smoking [19], and the risk of type 2 diabetes mellitus [20]. In addition, BMI, cigarette smoking and type 2 diabetes mellitus have been associated with a wide range of gastrointestinal diseases in our previous MR studies [21–23]. Thus, we considered these three factors potential mediators. The genetic instrumental variables of BMI, smoking initiation, and type 2 diabetes mellitus were respectively extracted from publicly available GWASs [24–26], and the detailed information can be found in Table S1. Independent (linkage disequilibrium *r*^*2*^ > 0.01) SNPs associated with BMI, smoking initiation, and type 2 diabetes at genome-wide significance threshold (*P* < 5 × 10^−8^) were selected as instrumental variables.

### Statistical analysis

SNPs were excluded if unavailable in outcome datasets or defined as ambiguous (i.e, palindromic SNPs with minor allele frequencies >0.42 and <0.58). The primary MR analysis was conducted by the inverse-variance weighted (IVW) method under a multiplicative random effects model. Assuming that all SNPs are valid instruments, the IVW method provides the most precise estimates. Estimates for each outcome from different sources were combined using the fixed-effects meta-analysis. Heterogeneity among estimates of SNPs was evaluated by Cochran’s *Q* value. To detect potential horizontal pleiotropy and examine the consistency of the associations, three sensitivity analyses including the weighted median [27], MR-Egger [28], and Mendelian randomization pleiotropy residual sum and outlier (MR-PRESSO) [29] analyses were performed. The weighted median method can provide consistent estimates if more than 50% of the weight in the analysis comes from valid genetic instruments [27]. MR-Egger regression provides an MR estimate with adjustment for horizontal pleiotropy detected by its intercept test [28]. MR-PRESSO method can detect SNP outliers with pleiotropic effects and provide an estimate identical to that from IVW after removal of these outliers [29]. The Benjamini-Hochberg method that controls the false discovery rate (FDR) was applied to correct for multiple testing. The association with a nominal *P* < 0.05 but Benjamini–Hochberg adjusted *P* > 0.05 was regarded suggestive and the association with a Benjamini–Hochberg adjusted *P* < 0.05 were deemed significant. All analyses were two-sided and performed using the TwoSampleMR [30], MendelianRandomization [27], and MRPRESSO [29] R packages in R software 4.1.2.

To investigate possible pathways linking depression to gastrointestinal diseases, we conducted a two-step MR analysis [31] to explore the mediation effects of BMI, cigarette smoking, and type 2 diabetes mellitus using multivariable MR analysis. The two-step MR analysis was only performed for significant MR associations in primary analysis. In detail, we first obtained the MR effect estimates for depression on each mediator using the IVW method. Then the multivariable MR was performed to estimate the effect of three mediators on risk of gastrointestinal diseases with adjustment for depression. These two estimates for each gastrointestinal disease were multiplied together to estimate the indirect effect of depression. Finally, the proportion of the total effect explained by the mediators was calculated through dividing the mediated effect by the total effect. We also performed the same multivariable MR analysis for outcomes with the nonsignificant associations to reveal the potential association of depression with gastrointestinal disease independent of the mediators.

## Results

**Fig. 2:**
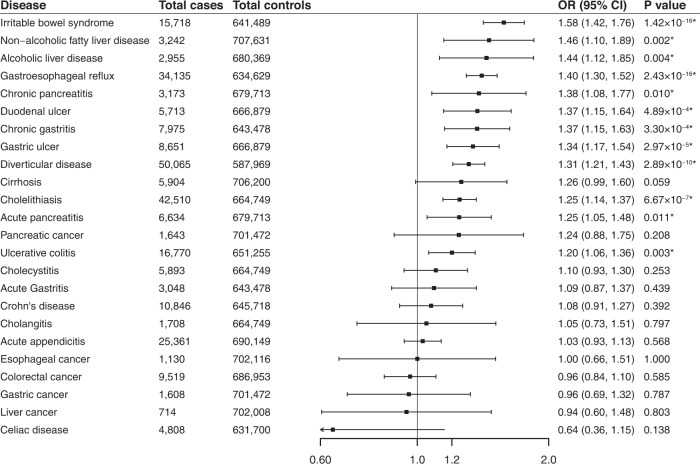
Associations of genetic liability to depression with 24 gastrointestinal diseases. *Significant association after multiple testing. The estimate of irritable bowel syndrome was meta-analysis by combining estimates from the UK Biobank study, the FinnGen study and the Genetic Epidemiology Research on Aging consortium; the estimates of Crohn’s disease and ulcerative colitis were meta-analysis by combining estimates from the UK Biobank study, the FinnGen study and the International Inflammatory Bowel Disease Genetics Consortium; the estimates of other gastrointestinal disease were meta-analysis by combining estimates from the UK Biobank study and the FinnGen study.

Genetic liability to depression was positively associated with 12 of the 24 studied gastrointestinal diseases and these associations remained after multiple comparison corrections (Fig. 2 and Table S4). In detail, genetic predisposition to depression was associated with higher odds of irritable bowel syndrome (OR 1.58; 95% CI: 1.42–1.76; *P* = 1.42 × 10^−16^), non-alcohol fatty liver disease (OR 1.46; 95% CI: 1.15–1.85; *P* = 0.002), alcoholic liver disease (OR 1.44; 95% CI: 1.12–1.85; *P* = 0.004), gastroesophageal reflux (OR 1.40; 95% CI: 1.30–1.52; *P* = 2.43 × 10^−16^), chronic pancreatitis (OR 1.38; 95% CI: 1.08–1.77; *P* = 0.01), duodenal ulcer (OR 1.37; 95% CI: 1.15–1.64; *P* = 4.89 × 10^−4^), chronic gastritis (OR 1.37; 95% CI: 1.15–1.63; *P* = 3.30 × 10^−4^), gastric ulcer (OR 1.34; 95% CI: 1.17–1.54; *P* = 2.97 × 10^−5^), diverticular disease (OR 1.31; 95% CI: 1.21–1.43; *P* = 2.89 × 10^−10^), cholelithiasis (OR 1.25; 95% CI: 1.14–1.37; *P* = 6.67 × 10^−7^), acute pancreatitis (OR 1.25; 95% CI: 1.05–1.48; *P* = 0.011, and ulcerative colitis (OR 1.20; 95% CI: 1.06–1.36; *P* = 0.003). The results of the sensitivity analysis were generally consistent (Table S5). The MR-Egger intercept tests found the indication of horizontal pleiotropy for diverticular disease in the UK Biobank study (P for MR-Egger intercept <0.05, Table S5) but not for any other outcomes in neither of sources. MR-PRESSO detected 1 to 2 outliers in the analysis for cholelithiasis; however, the association persisted after removal of the SNPs (Table S5).

Genetic liability to depression was significantly associated with higher levels of BMI and the higher risk of smoking initiation and type 2 diabetes mellitus (Table S6). Table 1 display the results of multivariable MR analyses and mediation effect of individual mediators and combinations of three mediators. We notice that BMI (49.28%) and combination of three mediators (53.54%) mediated quite a proportion of effect of depression on non-alcoholic fatty liver disease. For the causal effect of depression on acute pancreatitis, the proportion mediated by smoking initiation was up to 45.02%.

**Table 1:** Estimates of depression on gastrointestinal diseases mediated by potential mediators.

Gastrointestinal diseases	Cases/Controls	Adjustment for BMI			Adjustment for smoking initiation			Adjustment for type 2 diabetes mellitus			Adjustment for body mass index, smoking initiation, and type 2 diabetes mellitus		
		OR (95%CI)	*P* value	Mediation effect (%)	OR (95% CI)	*P* value	Mediation effect (%)	OR (95% CI)	*P* value	Mediation effect (%)	OR (95% CI)	*P* value	Mediation effect (%)
Gastroesophageal reflux	34,135/634,629	1.31 (1.20, 1.44)	7.31E-09	3.79%	1.32 (1.15, 1.52)	8.67E-05	7.24%	1.39 (1.24, 1.56)	3.32E-08	2.39%	1.30 (1.19, 1.42)	1.68E-08	10.76%
Gastric ulcer	8651/666,879	1.24 (1.04, 1.47)	0.017	6.57%	1.34 (1.06, 1.70)	0.014	16.34%	1.33 (1.09, 1.64)	7.83E-04	4.60%	1.28 (1.08, 1.52)	0.004	18.34%
Duodenal ulcer	5713/666,879	1.20 (0.96, 1.48)	0.102	11.51%	1.12 (0.82, 1.54)	0.481	19.67%	1.26 (0.94, 1.68)	0.118	5.34%	1.19 (0.97, 1.47)	0.101	23.69%
Chronic gastritis	7975/643,478	1.16 (1.02, 1.33)	0.028	7.08%	1.20 (0.99, 1.45)	0.059	13.45%	1.30 (1.10, 1.55)	0.003	5.30%	1.15 (1.00, 1.31)	0.043	29.14%
Irritable bowel disease	12,601/587,969	1.29 (1.22, 1.36)	3.89E-20	8.82%	1.28 (1.18, 1.38)	6.94E-10	14.92%	1.30 (1.21, 1.40)	1.94E-12	4.55%	1.27 (1.20, 1.34)	7.10E-18	20.64%
Diverticular disease	50,065/587,969	1.27 (1.15, 1.40)	1.05E-06	4.26%	1.23 (1.07, 1.41)	0.003	9.99%	1.22 (1.08, 1.38)	0.002	2.75%	1.26 (1.15, 1.39)	1.21E-06	10.30%
Ulcerative colitis	9,802/630,791	1.15 (1.04, 1.27)	0.005	−4.93%	1.23 (1.05, 1.44)	0.011	−11.21%	1.12 (0.97, 1.28)	0.115	7.12%	1.19 (1.08, 1.32)	6.41E-04	3.42%
Non-alcoholic fatty liver disease	3,242/707,631	1.05 (0.78, 1.42)	0.735	49.28%	1.27 (0.87, 1.85)	0.213	7.89%	1.35 (0.84, 2.18)	0.220	17.70%	1.09 (0.80, 1.49)	0.597	53.54%
Alcoholic liver disease	2,955/680,369	1.32 (1.03, 1.69)	0.027	4.48%	1.26 (0.91, 1.74)	0.157	34.13%	1.28 (0.89, 1.83)	0.183	3.27%	1.21 (0.94, 1.56)	0.131	36.43%
Cholelithiasis	5,893/664,749	1.13 (1.03, 1.24)	0.007	23.46%	1.18 (1.05, 1.33)	0.005	3.18%	1.17 (1.01, 1.37)	0.041	9.30%	1.14 (1.03, 1.25)	0.008	18.06%
Acute pancreatitis	6,634/679,713	1.18 (0.99, 1.40)	0.068	11.01%	1.07 (0.86, 1.34)	0.532	45.02%	1.12 (0.91, 1.37)	0.295	7.36%	1.13 (0.95, 1.34)	0.152	38.08%
Chronic pancreatitis	3,173/679,713	1.41 (1.16, 1.72)	7.18E-04	0.91%	1.10 (0.86, 1.40)	0.435	26.79%	0.97 (0.88, 1.08)	0.585	−2.70%	1.02 (0.95, 1.11)	0.557	44.07%

*OR* odds radio, *CI* confidence interval.

In multivariable MR analysis adjusting for genetically predicted BMI, genetic liability to depression was significant associated with increased risk of acute gastritis (OR 1.49; 95% CI: 1.22–1.81; *P* = 7.35 × 10^−5^) and cirrhosis (OR 1.28; 95% CI: 1.10–1.48; *P* = 0.001). When adjusting for genetically predicted smoking initiation (OR 1.35; 95% CI: 1.11–1.65; *P* = 0.003) and type 2 diabetes mellitus (OR 1.40; 95% CI: 1.11–1.77; *P* = 0.005) separately, genetic liability to depression was positively associated with cirrhosis (Table S7).

## Discussion

We performed a comprehensive MR investigation on the associations of genetic liability to depression with 24 gastrointestinal diseases. We found that genetic liability to depression was associated with the increased risk of 12 gastrointestinal diseases. Multivariable MR analyses indicated that association between depression and non-alcoholic fatty liver disease were substantially mediated by BMI. Genetically prediction to smoking initiation mediated half of effect of depression on acute pancreatitis.

The current MR investigation corroborated previous epidemiological studies’ findings that depression was associated with an increased risk of irritable bowel syndrome [6], non-alcoholic fatty liver disease [32], gastroesophageal reflux [7], gastric ulcer and duodenal ulcer [8]. However, previous evidence on the association between depression and alcoholic liver disease is inconclusive. A cross-sectional study including 398 patients with alcoholic liver disease found that depression was not associated with alcoholic liver disease [33]; however, another study found that the prevalence and incidence of alcoholic liver disease were higher in patients with depression [34]. Our MR analysis found a positive association of genetic liability to depression with alcoholic liver disease. The unmeasured confounding and relatively small sample size might account for the discrepancy. An MR study found that genetic liability depression proxied by 19 SNPs associate with depression at *P* < 5 × 10^−6^ was positively associated with both Crohn’s disease and ulcerative colitis risk [35]. Our study replicated the positive association between depression and ulcerative colitis. However, a neutral association for Crohn’s disease was identified in this study with a larger sample size and updated instruments. The discrepancy may be caused by undetected horizontal pleiotropy introduced by using SNPs associated with the exposure at a relaxed threshold. Besides, the different definitions of depression in the original GWAS where the instruments were extracted may also attribute to this discrepancy. A phenome-wide MR study in the UK Biobank revealed that major depressive disorder was associated with increased risks of gastroesophageal reflux disease, non-infectious gastroenteritis, and gastrointestinal hemorrhage [12], which supports our findings. Our MR investigation refined the gastrointestinal classification and provided novel findings for gastric ulcer, duodenal ulcer, and chronic gastritis. The associations of depression with acute and chronic pancreatitis were also novel findings, which need to be verified.

Previous studies have suggested that nicotine in tobacco may have certain beneficial effects on patients with depression, which included relief of stress and depressive affect, and feeling pleasurable sensations [36]. Besides, nicotine cessation may result in withdrawal symptoms such as anhedonia and depression [37]. However, the health benefits of quitting smoking are immediate and long-lasting and evidence from current study showed that smoking mediated part of effect of depression on gastrointestinal diseases. Considering the complicated relationship between smoking and depression, the risks and benefit assessment of quitting was required for depression patients. The current study also uncovered that genetic liability to depression was associated increased risks of acute gastritis and cirrhosis. This suggested that the depression may have no effects on these diseases if the depression does not result in BMI increase.

The current study quantified the mediation effects of BMI, smoking initiation, and type 2 diabetes in the associations between genetic liability to depression and gastrointestinal disease risk. Our findings suggest that the prevention strategies on these three mediators might partly counteract the detrimental effects of depression on many gastrointestinal diseases. The findings of our MR investigation have implications for public health policy that psychologists should pay more attention to gastrointestinal disease screening and prevention for patients with depressive disorder.

In addition to mediated pathways by BMI, smoking, and diabetes, several biological mechanisms might explain the direct effect of depression on gastrointestinal disease. Autonomic dysfunction in depression results in dysfunction of gastric acid secretion [38], which leads to gastroesophageal reflux and peptic ulcer disease [39]. Chronic stress activates the neuroendocrine response to produce cortisol, which disturbs the balance of gut microflora and leads to bowel inflammation [40]. Imbalance in the gut microbiota composition may contribute to intestinal as well as extraintestinal diseases via perturbed microbiota that produces multiple substances including neuropeptides, hormones, and short-chain fatty acids [41].

The major strength of the current study is MR design, which minimizes biases caused by residual confounding and reverse causality. In addition, we examined the associations in two or more independent sources. The results from these data sources were generally consistent, which makes it unlikely that the observed associations were caused by chance. We explored the mediating pathways by conducting multivariable MR analysis, which deepened the mechanistic understandings and provided evidence supports for prevention strategies.

This study has limitations that warrant acknowledgment. A major limitation is that we could not completely rule out horizontal pleiotropy, which means genetic variants influence the outcome not or not only through the exposure. However, we performed several sensitivity analyses and found the associations were stable. In addition, we observed limited data of pleiotropy detected by MR-Egger and MR-PRESSO analyses. Given that MR utilizes genetic variants that cannot be modified to proxy the exposure, the design reflects the cumulative lifelong effect. Nevertheless, depression has a dynamic natural course in which symptoms partially remit over time even without treatment. Thus, the results might not be directly compared with observational findings and applicable to clinical practice. Another limitation is that we did not examine the bidirectional associations between depression and gastrointestinal diseases due to the lack of instrumental variables for all gastrointestinal diseases. In addition, the UK Biobank study was included in both the exposure and outcome datasets, which might bias MR estimates toward the observational associations. However, most associations were replicated in the FinnGen study, which indicated that the bias caused by sample overlap should be minimal. Finally, it’s important to note that our study was conducted in the individuals of European ancestry, which might limit the generalizability of our findings to other populations.

In conclusion, our MR investigation provided evidence that genetic liability to depression was associated with increased risks of 12 gastrointestinal disease outcomes. BMI, smoking, and type 2 diabetes mellitus appeared to mediate many of these associations.

## Supplementary information

Table S1

Table S2

Table S3

Table S4

Table S5

Table S6

Table S7

## Data Availability

All data analyzed in this study can be obtained by a reasonable request to corresponding authors.

## Code availability

Codes used in current study are available from the corresponding author on reasonable request.
